# Syntheses and structures of *N*-(2-fluoro­phen­yl)-2-oxo-2*H*-chromene-3-carboxamide and *N*-[4-(methyl­sulfon­yl)phen­yl]-2-oxo-2*H*-chromene-3-carboxamide

**DOI:** 10.1107/S2056989026001180

**Published:** 2026-02-10

**Authors:** M. Sunithakumari, H. C. Devarajegowda, M. U. Gagan, V. Dwarakanath, H. T. Srinivasa, B. S. Palakshamurthy

**Affiliations:** a Department of Physics Yuvaraja's College University of Mysore,Mysore 570005 Karnataka India; b Department of Biotechnology, UCS Tumkur University Tumkur Karnataka-572103 India; c Raman Research Institute, C V Raman Avenue Sadashivanagar Bangalore Karnataka-560080 India; d Department of PG Studies and Research in Physics Albert Einstein Block UCS Tumkur University, Tumkur Karnataka-572103 India; University of Aberdeen, United Kingdom

**Keywords:** crystal structure, 2-oxo-2*H*-chromene, Hirshfeld surface analysis, anti-bacterial studies

## Abstract

In the first title compound, C_16_H_10_FNO_3_ (**I**), the dihedral angle between the 2-oxo-2*H*-chromene ring system and the 2-fluoro­phenyl ring is 0.73 (16)°. In the second title compound, C_17_H_13_NO_5_S (**II**), the corresponding angle is 12.44 (2)°. Compound (**I**) features a bifurcated intra­molecular N—H⋯(O,F) hydrogen bond, whereas (**II**) displays an N—H⋯O hydrogen bond. In the crystal of (**I**), the mol­ecules are connected through pairwise C—H⋯O hydrogen bonds forming an inversion dimer generating an *R*_2_^2^(14) motif, whereas in (**II**), C—H⋯O hydrogen bonds generate an *R*_2_^2^(8) motif. The packing for (**I**) also features a C—H⋯F contact, generating an *S*(6) chain along [001]. The major contributions to the Hirshfeld surface of (**I**) are from H⋯H (30.0%), O⋯H/H⋯O (21.0%), C⋯H/H⋯C (15.9%), C⋯C (12.5%), F⋯H/H⋯F (10.8%) and O⋯C/C⋯O (5.0%) contacts while those in (**II**) are from H⋯H (28.0%), O⋯H/H⋯O (36.7%), C⋯H/H⋯C (19.8%), C⋯C (5.6%), and O⋯C/C⋯O (6.9%) contacts. Compound (**II**) demonstrated moderate to good anti-bacterial activity with MIC values of 25 µg ml^−1^ against *S. aureus* and 15 µg ml^−1^ against *E. coli.*

## Abstract 2

Two 2-oxo-2*H*-chromene derivatives were synthesized by acid–aniline coupling reactions. These two compounds were subjected to SXRD and Hirshfeld surface analysis to explore their inter­molecular inter­actions.

## 1. Chemical context

The 2-oxo-2*H*-chromene scaffold is associated with a wide range of biological activities, including anti­cancer (Phutdhawong *et al.*, 2021 ▸; Sunitha Kumari *et al.*, 2025 ▸), anti-inflammatory (Melagraki *et al.*, 2009 ▸), anti­tubercular (Rana *et al.*, 2025 ▸) and anti­microbial properties (Sangani *et al.*, 2013 ▸). Recent studies have demonstrated that conjugated 2-oxo-2*H*-chromene derivatives exhibit considerable anti­microbial potential (Lata *et al.*, 2024 ▸). Efficient synthetic approaches, such as one-pot and multicomponent reactions, have enabled the development of structurally diverse coumarin derivatives (Eshghi *et al.*, 2021 ▸). Several investigations have reported the effectiveness of these compounds against pathogenic microorganisms associated with wound infections, often displaying advantages over conventional anti­biotics due to their distinct modes of action (Latha Rani *et al.*, 2016 ▸). Among these derivatives, 2-oxo-2*H*-chromene-3-carboxamides have attracted particular attention owing to their potential as anti-*Helicobacter pylori* agents (Chimenti *et al.*, 2007 ▸). Carboxamide frameworks are widely employed in pharmaceutical design and are known to exhibit anti­bacterial and anti­oxidant properties, highlighting their importance in drug-discovery programmes (Gadhave *et al.*, 2022 ▸). In addition, phenyl-substituted coumarins have been reported to display anti­microbial activity comparable to that of kanamycin (Nayak *et al.*, 2015 ▸).
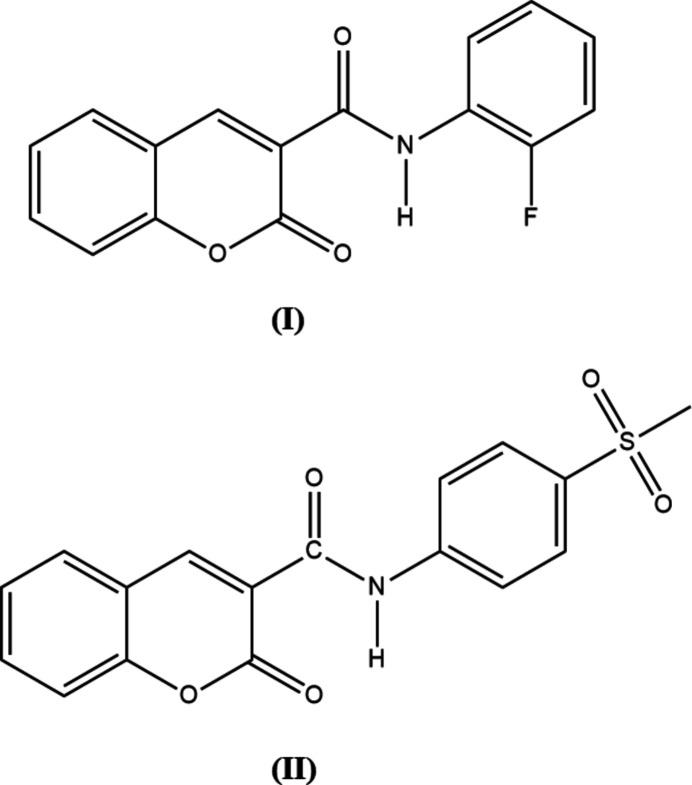


The incorporation of halogen and sulfonyl substituents has been shown to enhance biological activity in some cases, including notable anti­cancer effects against breast cancer cell lines (Althobaiti *et al.*, 2025 ▸). Sulfonyl-containing heterocycles, such as quinazoline analogues, have also exhibited promising anti­cancer and anti-inflammatory activities, underscoring the therapeutic relevance of sulfonyl-functionalized scaffolds (Venkatesan *et al.*, 2024 ▸). Amide bond formation mediated by 1-[bis­(di­methyl­amino)­methyl­ene]-1*H*-1,2,3-triazolo[4,5-*b*]pyridinium 3-oxide hexa­fluoro­phosphate (HATU) in the presence of tri­ethyl­amine (TEA) is a reliable and widely used synthetic method. Coupling halogenated phenyl­aniline and other rigid aromatic moieties with the 2-oxo-2*H*-chromene core has yielded derivatives with enhanced anti­bacterial activity. In continuation of our ongoing studies on these systems, we report herein the synthesis and crystal structures of the title compounds C_16_H_10_FNO_3_ (**I**) and C_17_H_13_NO_5_S (**II**). Hirshfeld surfaces are computed for both compounds and preliminary anti-bacterial data are reported for (**II**).

## 2. Structural commentary

**Figure 1:**
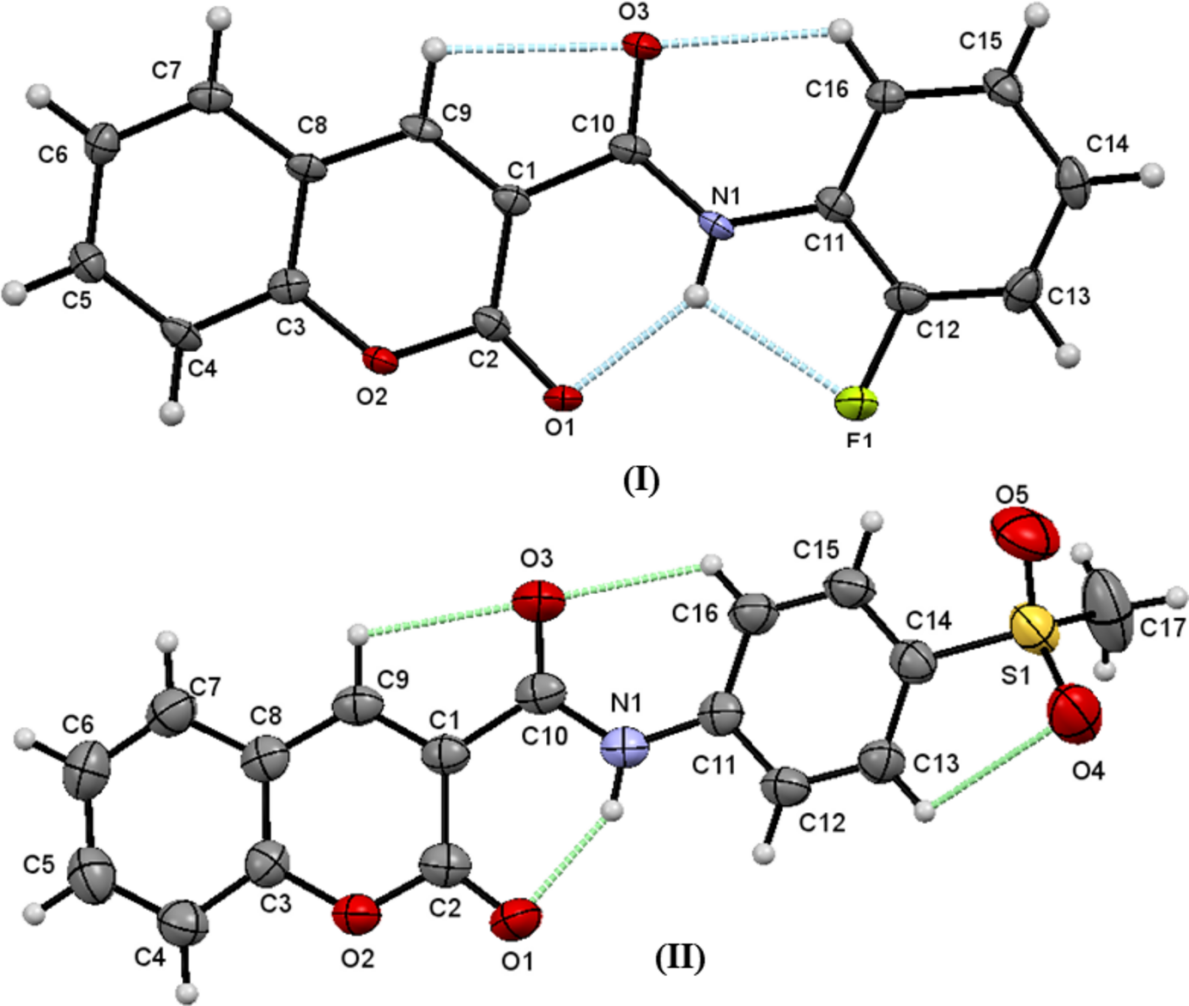
The mol­ecular structures of (**I**) and (**II**) showing 50% probability ellipsoids with intra­molecular hydrogen bonds and short contacts shown as blue dashed lines.

In compound (**I**), the dihedral angle between the C1–C9/O1/O2 2-oxo-2*H*-chromene ring system and the C11–C16 aromatic ring of the 2-fluoro­phenyl moiety is 0.73 (16)°: the mol­ecule is approximately planar with an r.m.s deviation of twenty fitted non-H atoms of 0.020 Å. In (**II**), the dihedral angle between the C1–C9/O1/O2 2-oxo-2*H*-chromene fused rings and the C11–C16 ring of the methyl­sulfonyl phenyl moiety is 12.44 (2)°. The C1—C10(O)—N1(H)—C11 torsion angles associated with the amide moiety of (**I**) and (**II**) are 179.9 (4) and −172.1 (3)°, respectively, indicating the expected *trans* conformation in each case. The bond angle for the linking C14(Ar)—S1—C17 bond in (**II**) of 104.63 (19)° correlates with a near perpendicular arrangement of the terminal methyl group with the aromatic ring phenyl moiety. Compound (**I**) features a bifurcated intra­molecular N—H⋯(O,F) hydrogen bond (Table 1 ▸), whereas (**II**) displays an intra­molecular N—H⋯O hydrogen bond (Table 2 ▸). Both (**I**) and (**II**) feature the same two short intra­molecular C—H⋯O contacts associated with atoms C9, C16 and O3 (Fig. 1 ▸). All in all, the solid-state conformations of (**I**) and (**II**) are very similar, with the same intra­molecular non-covalent inter­actions.

## 3. Supra­molecular features

**Figure 2:**
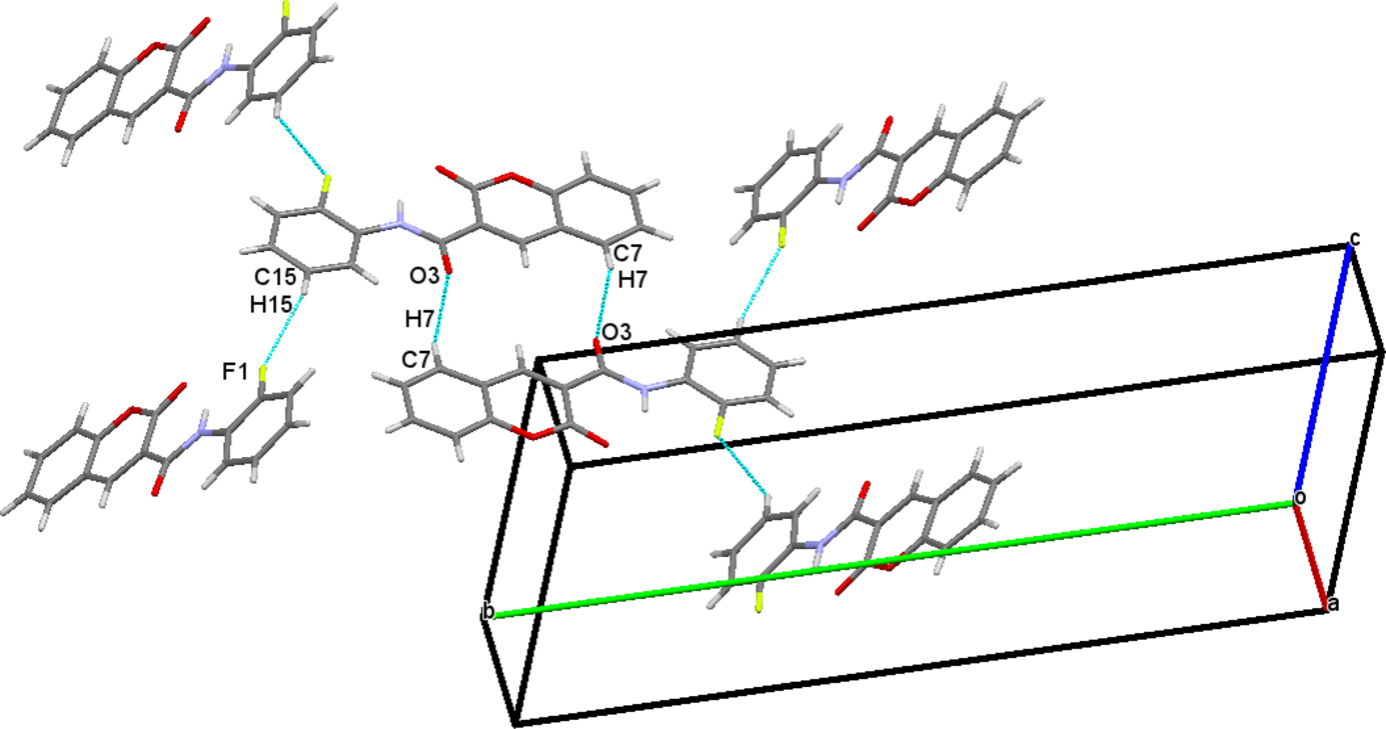
The packing diagram for (**I**): C—H⋯O hydrogen bonds generating an 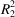
(14) motif and weak C—H⋯F inter­actions generating an *S*(6) chain along [001] are shown as dashed lines.

**Figure 3:**
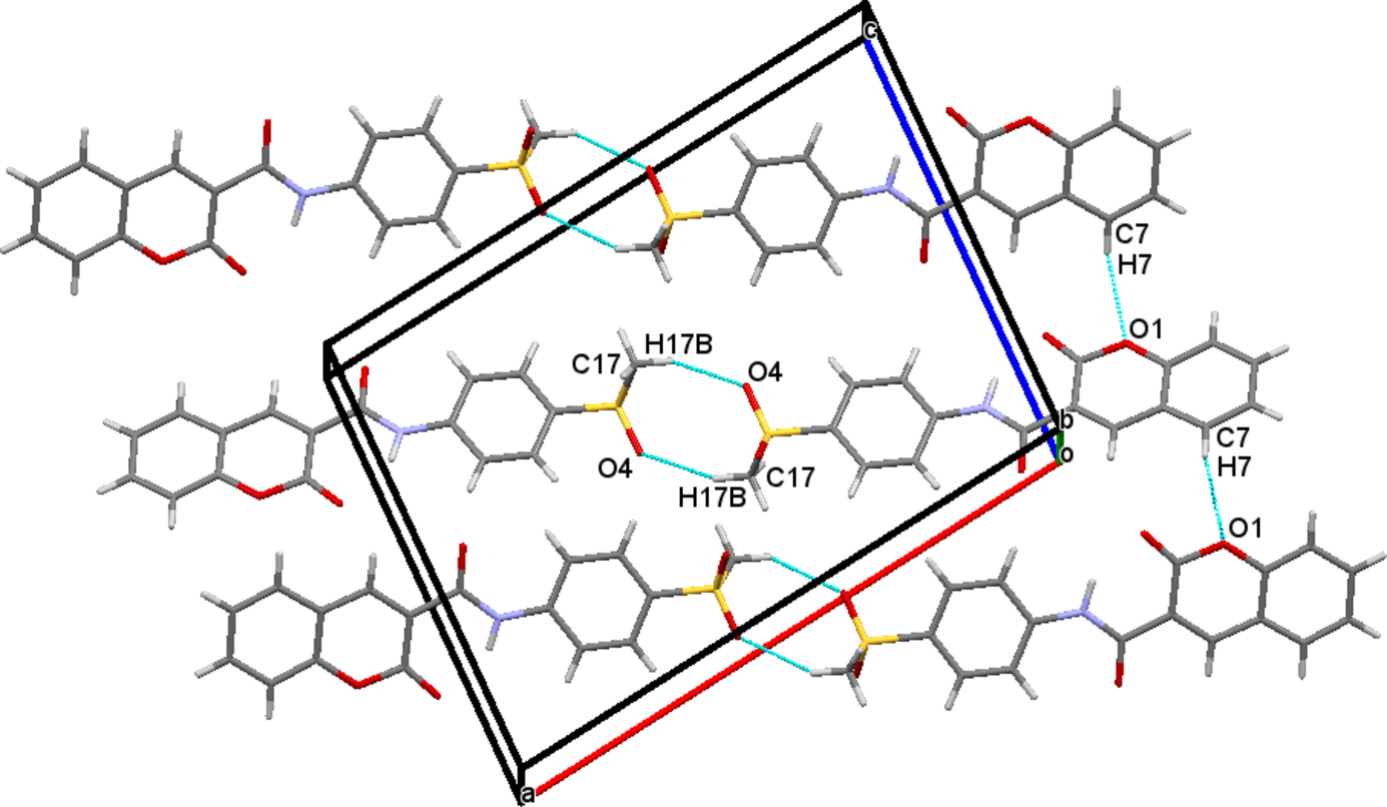
The packing diagram for (**II**): C—H⋯O hydrogen bonds generating 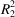
(8) motifs and *S*(5) chains along [001] are shown as dashed lines.

In the extended structure of (**I**), inter­molecular C—H⋯O and C—H⋯F hydrogen bonds are observed (Table 1 ▸). The mol­ecules are connected through pairwise C7—H7⋯O3 hydrogen bonds, forming an inversion dimer generating an 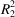
(14) motif (Fig. 2 ▸). In addition, weak C15—H15⋯F1 inter­actions generate *S*(6) chains propagating along [001]. In (**II**), C7—H7⋯O1, C17—H17*A*⋯O5 and C17—H17*B*⋯O4 inter­molecular inter­actions are observed (Table 2 ▸). Among these, C7—H7⋯O1 forms an *S*(5) chain propagating along the [001] direction and C17—H17*B*⋯O4 generates an 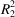
(8) motif by connecting two mol­ecules into an inversion dimer (Fig. 3 ▸).

**Figure 4:**
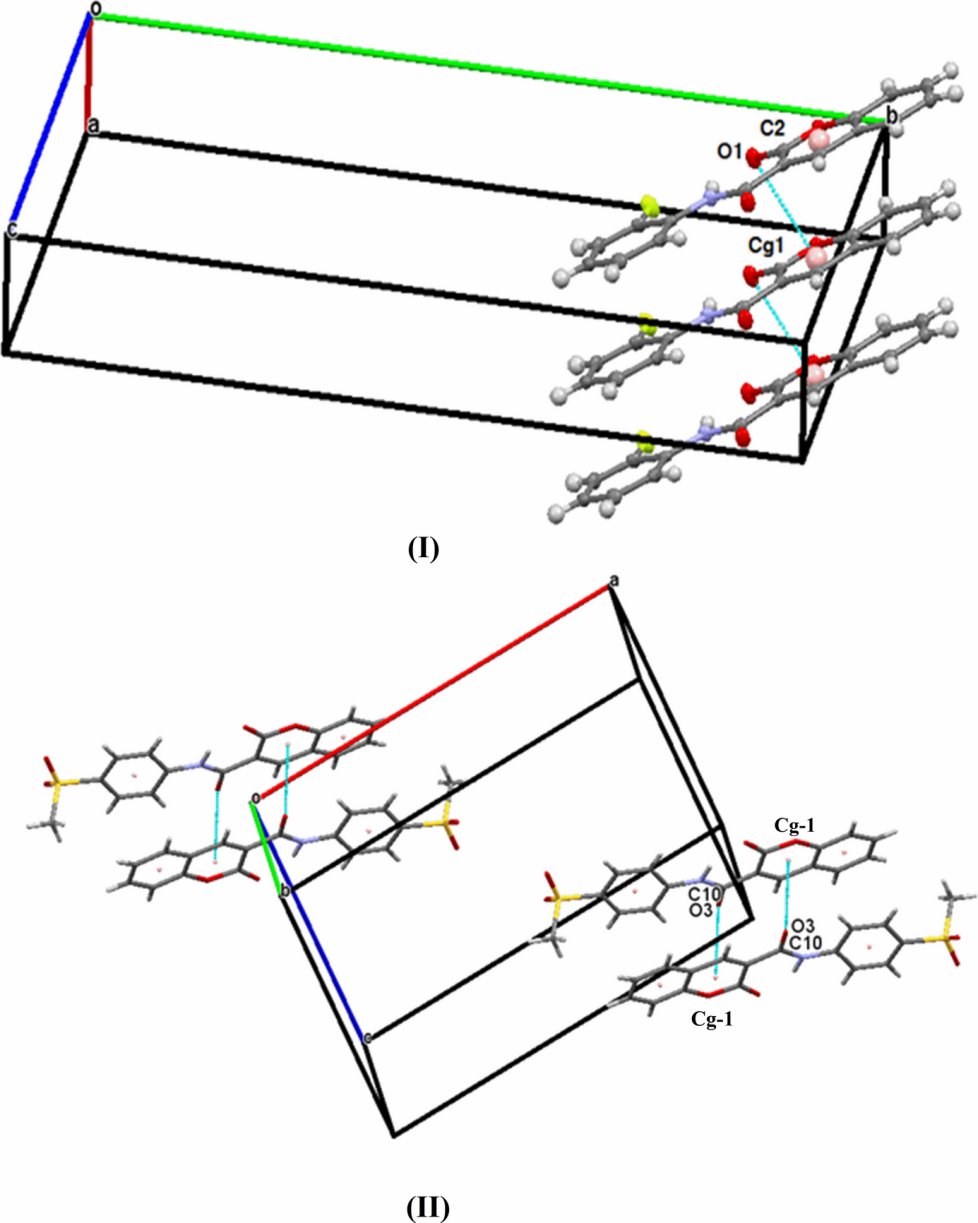
The partial packing of (**I**) and (**II**) showing the C=O⋯π short contacts.

**Figure 5:**
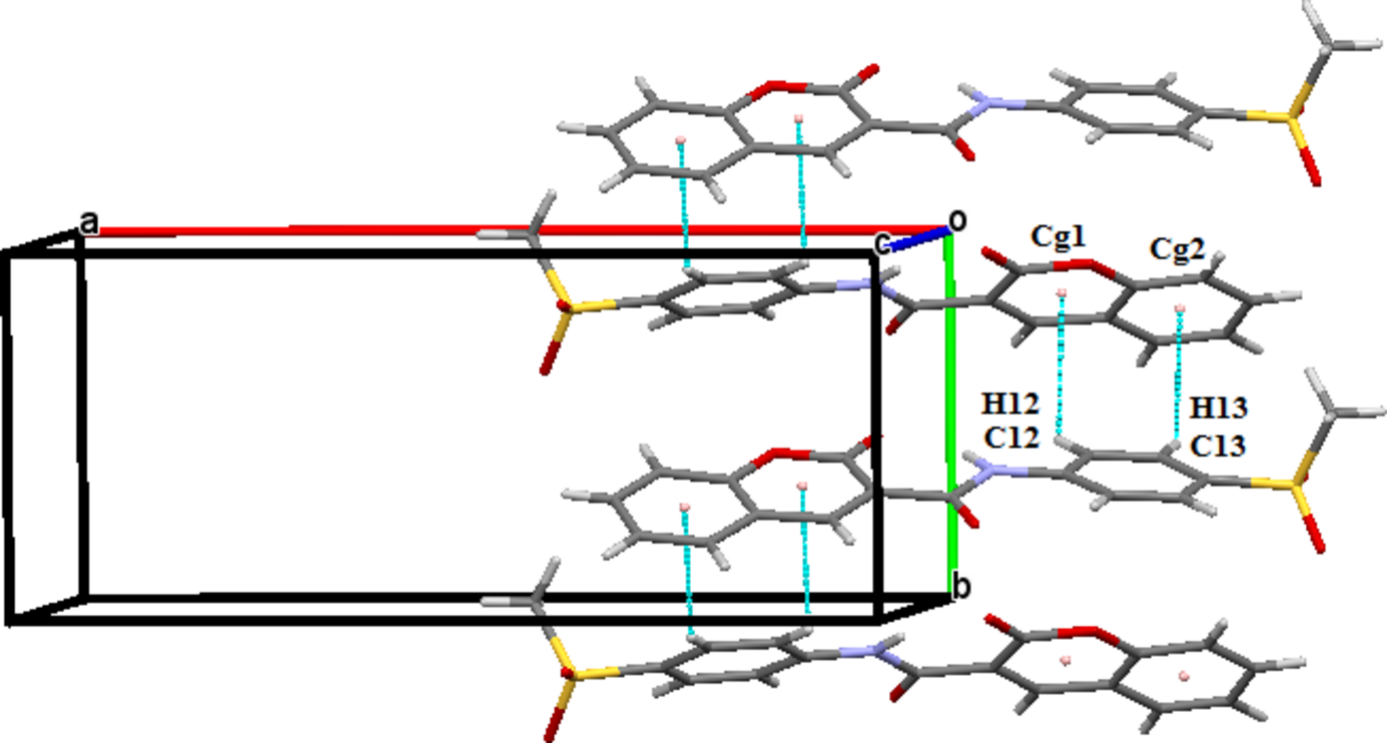
The partial packing of (**II**) indicating the C—H⋯π inter­actions.

**Figure 6:**
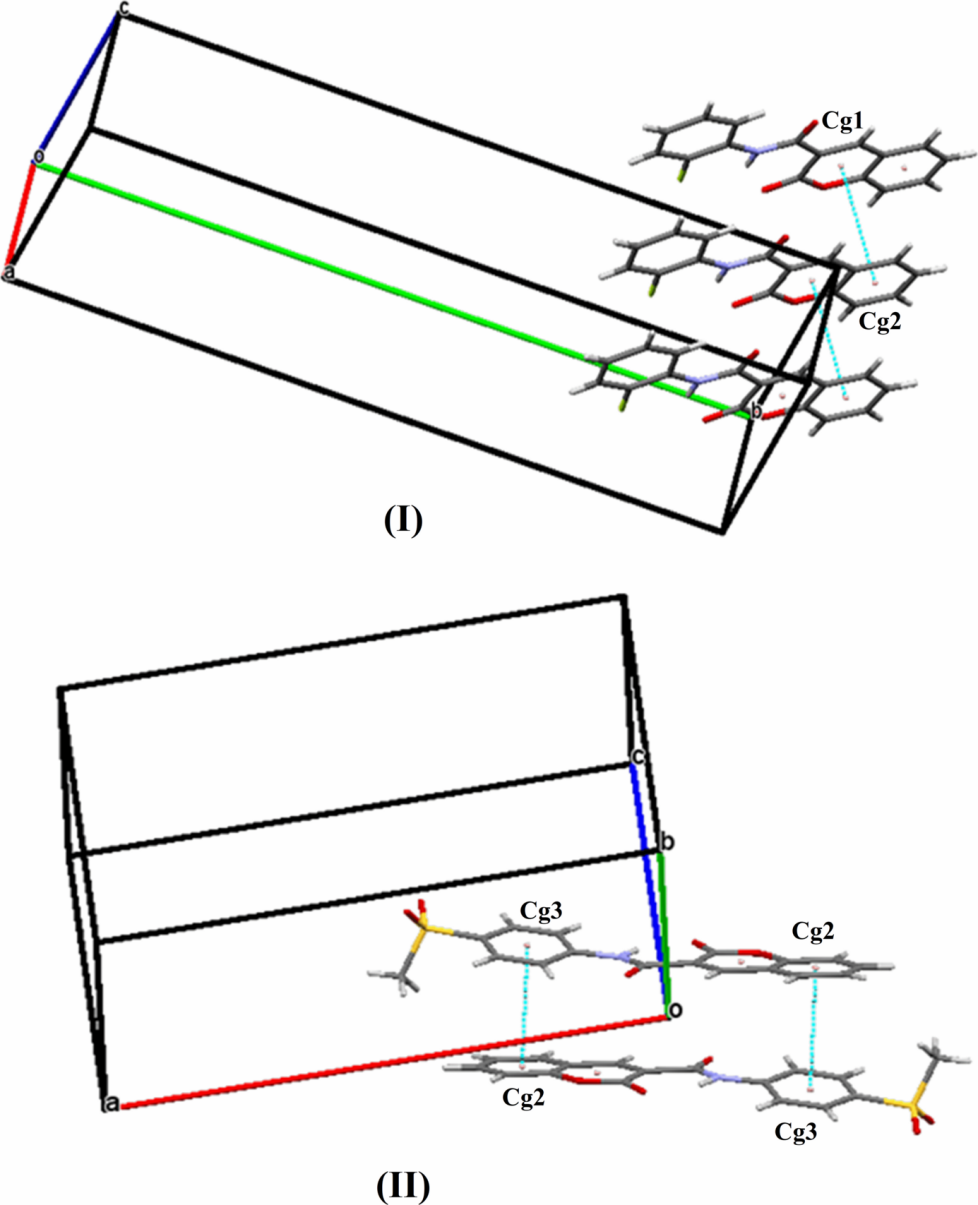
The partial packing of (**I**) and (**II**) indicating π–π stacking.

Both structures feature a C10=O3⋯*Cg*1 close contact between the carbonyl group and the heterocyclic ring of the coumarin moiety (Fig. 4 ▸) with an O⋯π separation of 3.383 (3) Å for (**I**) and 3.465 (3) Å for (**II**) compared to a van der Waals separation of 3.32 Å. Furthermore, the packing of (**II**) is consolidated by two C12—H12⋯*Cg*2, C13—H13⋯*Cg*1 inter­actions as shown in Fig. 5 ▸. In addition to these, the packing for (**I**) and (**II**) is consolidated by aromatic π⋯π stacking with a centroid–centroid distance, *Cg*1⋯*Cg*2 = 3.662 (2) Å in (**I**) and *Cg*2⋯*Cg*3 = 3.917 (2) Å in (**II**) (Fig. 6 ▸) where *Cg*1 and *Cg*2 are the centroids of the O2/C2/C1/C9/C8/C3 and C3–C8 coumarin rings in (**I**) or (**II**) and *Cg*3 is centroid of the C11–C16 ring in (**II**).

## 4. Database survey

A search of the Cambridge Structural Database (CSD, updated to July 2025; Groom *et al.*, 2016 ▸) for structures containing a 2-oxo-2*H*-chromene-3-carboxamide fragment yielded more than thirty entries, of which four are closely related to compounds (**I**) and (**II**). In the structures with refcodes DISYIP (Maldonado-Domínguez *et al.*, 2014 ▸), IPODIC (Gomes *et al.*, 2016 ▸ ▸) and IPODOI (Gomes *et al.*, 2016 ▸ ▸), the dihedral angles between the 2-oxo-2*H*-chromene core and the substituted phenyl rings are less than 2°, indicating near coplanarity, comparable with that observed in (**I**) and (**II**). In these structures, the amide linkage adopts a *trans* conformation, with torsion angles close to 180°, consistent with those found in the title compounds. By contrast, in *N*-(2,6-di­methyl­phen­yl)-2-oxo-2*H*-chromene-3-carboxamide (KEVGUQ; Yu *et al.*, 2018 ▸), the dihedral angle between the chromene system and the dimethyl-substituted phenyl ring deviates significantly from planarity, approaching orthogonality, which can be attributed to steric effects arising from the two methyl substituents. In addition, three structures containing an *N*-(4-methyl­sulfon­yl)phenyl fragment [FUGKIB (Ghosh *et al.*, 2000 ▸), HIZMIP (Tian *et al.*, 2019 ▸) and MOTJEK (Daszkiewicz *et al.*, 2002 ▸)] were identified. In these compounds, the geometry around the C(ar­yl)—S—C bond indicates a perpendicular orientation of the methyl­sulfonyl group relative to the phenyl ring, closely resembling that observed in compound (**II**). Overall, these observations indicate that mol­ecules incorporating the 2-oxo-2*H*-chromene-3-carboxamide moiety preferentially adopt a *trans*-amide geometry, consistent with the conformations observed in (**I**) and (**II**).

## 5. Hirshfeld surface analysis

**Figure 7:**
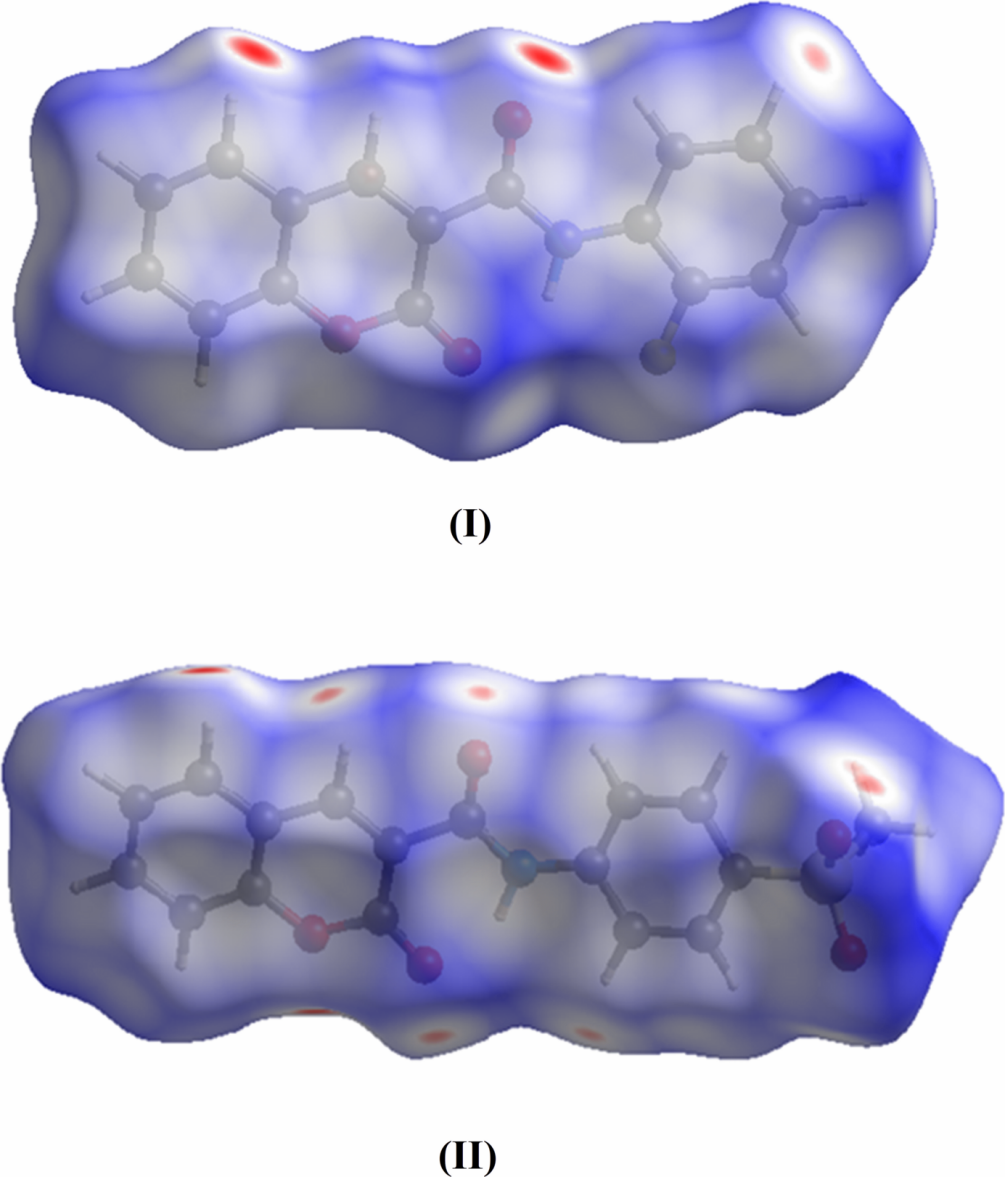
View of the three-dimensional Hirshfeld surfaces of (**I**) and (**II**) plotted over *d*_norm_.

**Figure 8:**
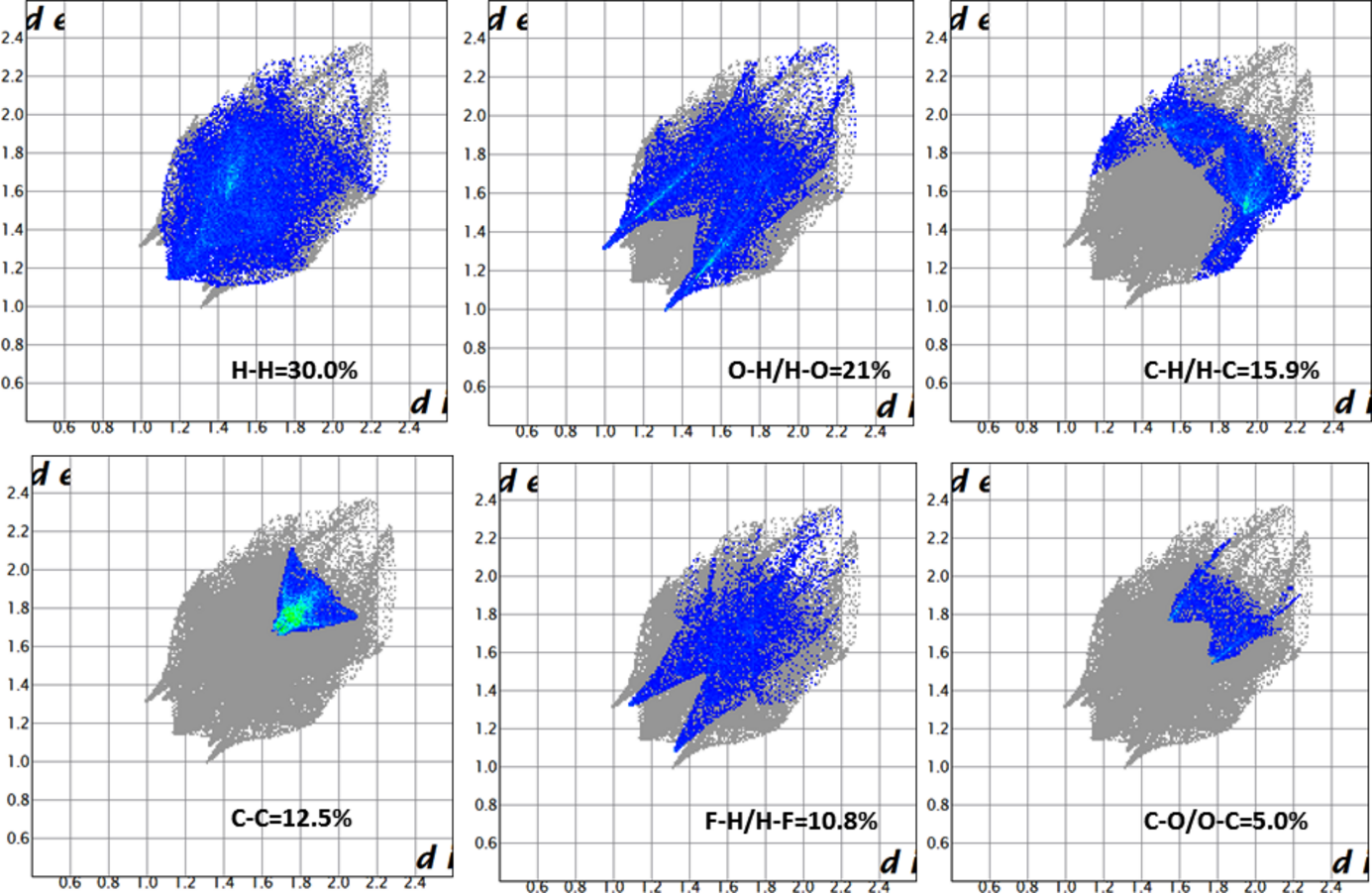
The two-dimensional fingerprint plots for compound (**I**), showing the different contact types.

**Figure 9:**
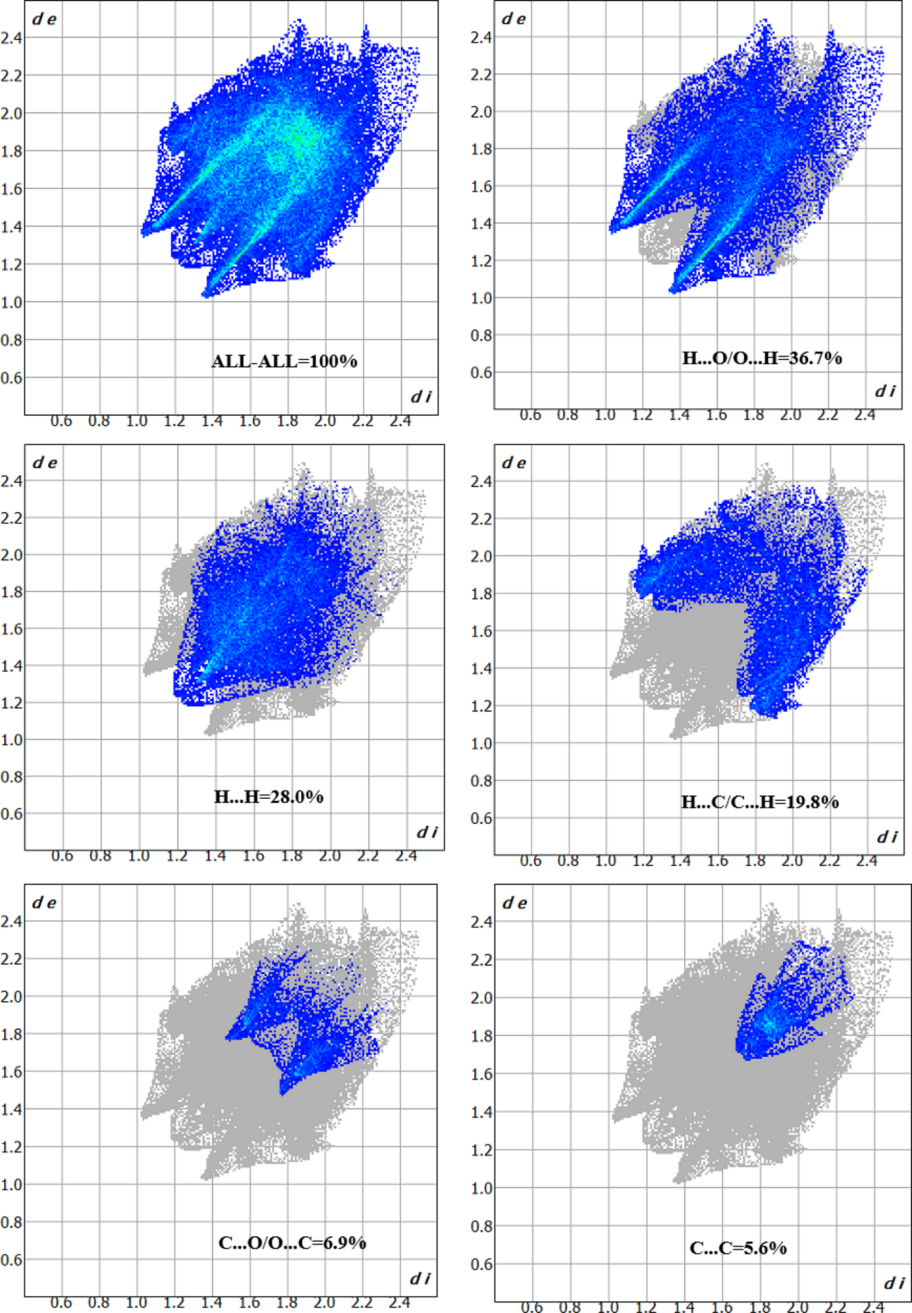
The two-dimensional fingerprint plots for compound (**II**), showing the different contact types.

A Hirshfeld surface analysis was carried out for (**I**) and (**II**) using *Crystal Explorer 17.5* (Spackman *et al.*, 2021 ▸) to further qu­antify the inter­molecular inter­actions listed in Tables 1 ▸ and 2 ▸. The three-dimensional Hirshfeld surfaces plotted over *d*_norm_ are shown in Fig. 7 ▸. The two-dimensional fingerprint plots for (**I**) indicate that the most important contributions for the Hirshfeld surface are from H⋯H (30.0%), H⋯O/O⋯H (21.0%), H⋯C/C⋯H (15.9%), C⋯C (12.5%), H⋯F/C⋯F (10.8%), and C⋯O/ O⋯C (5%) contacts as shown in Fig. 8 ▸. Similarly the fingerprint plots for (**II**) show the important contributions for the Hirshfeld surface are from H⋯H (28.0%), O⋯H/H⋯O (36.7%), C⋯H/H⋯C (19.8%), C⋯C (5.6%) and O⋯C/C⋯O (6.9%) contacts are shown in Fig. 9 ▸. Thus, the percentage of O⋯H/H⋯O contacts for (**II**) is substanti­ally higher than for (**I**), which possibly correlates with the ‘extra’ O atoms in the methyl­sulfonate group in the former and their role as inter­molecular hydrogen-bond acceptors (Table 2 ▸).

## 6. Anti-bacterial activities

The anti-bacterial efficacy of compound (**II**) was evaluated against gram-positive (*Staphylococcus aureus*) and gram-negative (*Escherichia coli*) bacterial strains by determining the minimum inhibitory concentration (MIC) (Boyanova *et al.*, 2005 ▸). Compound (**II**) exhibited moderate to good anti-bacterial activity, with MIC values of 25 µg ml^−1^ against *S. aureus* and 15 µg ml^−1^ against *E. coli*, indicating slightly enhanced activity towards the gram-negative strain. The estimated error for these measurements is ±1 µg ml^−1^. Compared with the standard anti­biotic ciprofloxacin, which exhibited potent activity against both tested organisms with MIC values of 15 µg ml^−1^, compound (**II**) showed approximately 1.7-fold lower potency against *S. aureus*; however, against *E. coli*, compound (**II**) demonstrated comparable efficacy, exhibiting an identical MIC value of 15 µg ml^−1^. These findings suggest that compound (**II**) possesses a promising anti­bacterial profile and might serve as a potential lead compound for further structure–activity relationship and mechanistic studies.

## 7. Synthesis and crystallization

**Figure 10:**
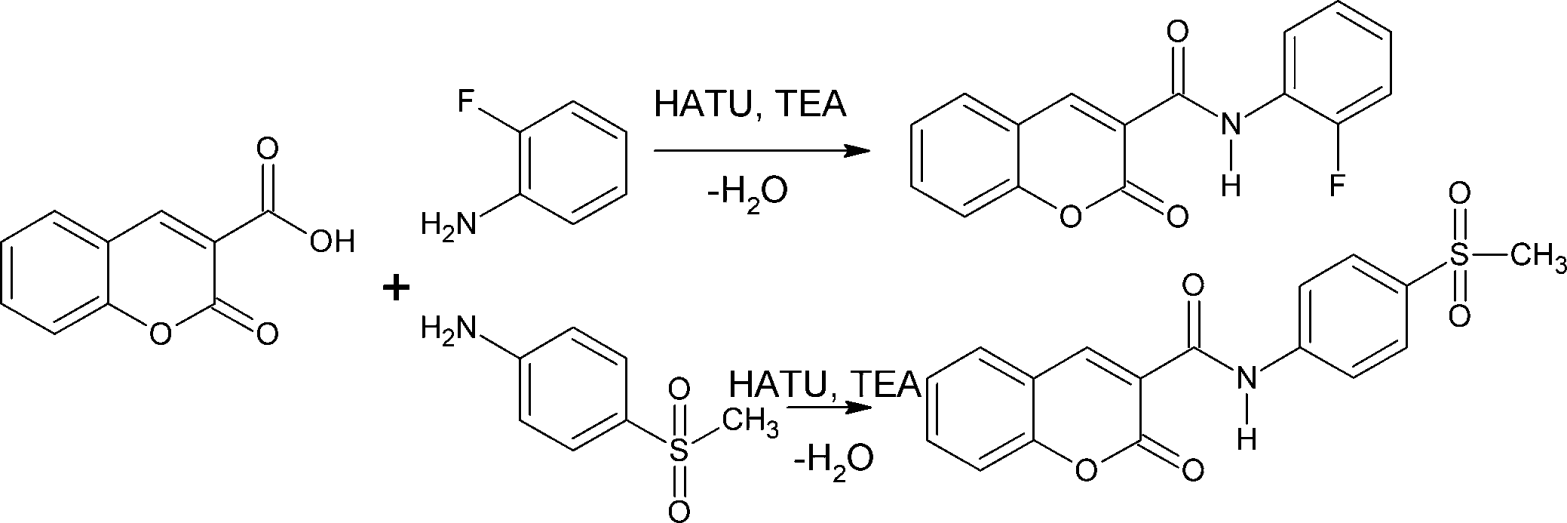
Synthesis schemes for (**I**) and (**II**).

A mixture of 2-oxo-2*H*-chromene-3-carb­oxy­lic acid (1.00 mmol), 2-fluoro­aniline (2.00 mmol) [for (**I**)] and 4-(methyl­sulfon­yl)aniline (2.00 mmol) [for (**II**)] and triethyl amine (TEA) (4.2 mmol) in aceto­nitrile (15 ml) was stirred at room temperature for 5 min. Then, 1-[bis­(di­methyl­amino)­methyl­ene]-1*H*-1,2,3-triazolo[4,5-*b*]pyridinium 3-oxide hexa­fluoro­phosphate (HATU) (5 mmol) was added in one portion, and the reaction was covered with a rubber septum (Fig. 10 ▸). After 24 h, the aceto­nitrile was removed *in vacuo*, and the residue was dissolved in di­chloro­methane (25 ml). The organic layer was washed with water (25 ml) and separated; the aqueous layer was extracted with di­chloro­methane. The combined organic layers were washed with brine, dried over magnesium sulfate, and concentrated under reduced pressure. The crude residue was purified by 60–120 mesh silica gel column chromatography (1:4 ethyl acetate/hexa­ne). Colourless prisms of (**I**) and (**II**) were recrystallized from ethyl acetate solution in each case. For (**I**): ^1^H NMR (500 MHz, CDCl_3_): δ (ppm) 10.83 (*s*, 1H, –CO—NH–), 8.45 (*s*, 1H, vinyl-H), 8.10–7.86 (*m*, 3H, Ar-H), 7.42–7.38 (*m*, 3H, Ar-H), 7.33–6.95 (*m*, 2H, Ar-H). M.p. 432 K; elemental analysis (%) calculated: C, 72.34; H, 3.93; F, 6.73; O 17.00; found C, 72.39; H, 3.95; F, 6.77%. For (**II**): ^1^H NMR (500 MHz, CDCl_3_: δ (ppm) 10.83 (*s*, 1H, –CO—NH–), 8.45 (*s*, 1H, vinyl-H), 7.86–7.66 (*m*, 4H, Ar-H), 7.42 (*m*, 2H, Ar-H), 6.86 (*m*, 2H, Ar-H), 3.41 (*s*, 3H, –CH_3_). M.p 458 K, elemental analysis (%) calculated: C, 59.47; H, 3.82; N, 4.08; O, 23.30; S, 9.34.; found C, 59.50; H, 3.87; N, 4.13; S, 9.39%.

## 8. Refinement

Crystal data, data collection and structure refinement details are summarized in Table 3 ▸. The hydrogen-atom positions were calculated geometrically (N—H = 0.86 Å; C—H = 0.93–0.96 Å) and refined using a riding model by applying the constraint *U*_iso_(H) = 1.2*U*_eq_(C, N) or 1.5*U*_eq_(methyl C).

## Supplementary Material

Crystal structure: contains datablock(s) I, II, global. DOI: 10.1107/S2056989026001180/hb8193sup1.cif

Structure factors: contains datablock(s) I. DOI: 10.1107/S2056989026001180/hb8193Isup2.hkl

Structure factors: contains datablock(s) II. DOI: 10.1107/S2056989026001180/hb8193IIsup3.hkl

Supporting information file. DOI: 10.1107/S2056989026001180/hb8193Isup4.cml

Supporting information file. DOI: 10.1107/S2056989026001180/hb8193IIsup5.cml

CCDC references: 2528508, 2528509

Additional supporting information:  crystallographic information; 3D view; checkCIF report

## Figures and Tables

**Table 1:** Hydrogen-bond geometry (Å, °) for (**I**)

*D*—H⋯*A*	*D*—H	H⋯*A*	*D*⋯*A*	*D*—H⋯*A*
N1—H1⋯O1	0.86	1.97	2.706 (4)	143
N1—H1⋯F1	0.86	2.25	2.647 (4)	108
C9—H9⋯O3	0.93	2.43	2.757 (5)	101
C16—H16⋯O3	0.93	2.34	2.918 (5)	120
C7—H7⋯O3^i^	0.93	2.47	3.326 (5)	154
C15—H15⋯F1^ii^	0.93	2.53	3.255 (5)	135

Symmetry codes: (i) 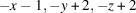; (ii) 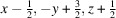.

**Table 2:** Hydrogen-bond geometry (Å, °) for (**II**) *Cg*1 and *Cg*2 are the centroids of the O2/C2/C1/C9/C8/C3 and C3/C4/C5/C6/C7/C8 rings.

*D*—H⋯*A*	*D*—H	H⋯*A*	*D*⋯*A*	*D*—H⋯*A*
N1—H1⋯O1	0.86 (4)	1.91 (4)	2.685 (4)	149 (3)
C9—H9⋯O3	0.93	2.45	2.771 (4)	100
C16—H16⋯O3	0.93	2.34	2.916 (4)	119
C13—H13⋯O4	0.93	2.51	2.890 (4)	105
C7—H7⋯O1^i^	0.93	2.82	3.624 (4)	146
C17—H17*A*⋯O5^ii^	0.96	2.53	3.163 (5)	123
C17—H17*B*⋯O4^iii^	0.96	2.47	3.293 (5)	144
C12—H12⋯*Cg*1^iv^	0.93	2.97	3.509 (4)	118
C13—H13⋯*Cg*2^iv^	0.93	2.86	3.522 (4)	129

Symmetry codes: (i) 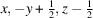; (ii) 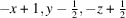; (iii) 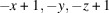; (iv) 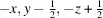.

**Table 3:** Experimental details

	(**I**)	(**II**)
Crystal data		
Chemical formula	C_16_H_10_FNO_3_	C_17_H_13_NO_5_S
*M*_r_	283.25	343.36
Crystal system, space group	Monoclinic, *P*2_1_/*n*	Monoclinic, *P*2_1_/*c*
Temperature (K)	296	289
*a*, *b*, *c* (Å)	3.7966 (2), 24.8289 (11), 12.9470 (7)	17.2099 (18), 7.0495 (7), 12.6535 (12)
β (°)	92.870 (2)	98.022 (3)
*V* (Å^3^)	1218.92 (11)	1520.1 (3)
*Z*	4	4
Radiation type	Mo *K*α	Mo *K*α
μ (mm^−1^)	0.12	0.24
Crystal size (mm)	0.32 × 0.25 × 0.21	0.38 × 0.30 × 0.25

Data collection		
Diffractometer	Bruker SMART APEXII CCD	Bruker SMART APEXII CCD
Absorption correction	Multi-scan (*SADABS*; Krause *et al.*, 2015 ▸)	Multi-scan (*SADABS*; Krause *et al.*, 2015 ▸)
*T*_min_, *T*_max_	0.963, 0.974	0.915, 0.941
No. of measured, independent and observed [*I* > 2σ(*I*)] reflections	22396, 2154, 1976	19137, 3042, 2177
*R*_int_	0.076	0.090
(sin θ/λ)_max_ (Å^−1^)	0.594	0.621

Refinement		
*R*[*F*^2^ > 2σ(*F*^2^)], *wR*(*F*^2^), *S*	0.093, 0.149, 1.28	0.064, 0.156, 1.05
No. of reflections	2154	3042
No. of parameters	190	221
H-atom treatment	H-atom parameters constrained	H atoms treated by a mixture of independent and constrained refinement
Δρ_max_, Δρ_min_ (e Å^−3^)	0.27, −0.35	0.26, −0.42

Computer programs: *APEX2* and *SAINT* (Bruker, 2017 ▸), *SHELXT* (Sheldrick, 2015*a* ▸), *SHELXL* (Sheldrick, 2015*b* ▸), *Mercury* (Macrae *et al.*, 2020 ▸) and *publCIF* (Westrip, 2010).

## supplementary crystallographic information

### N-(2-Fluorophenyl)-2-oxo-2H-chromene-3-carboxamide (I) Crystal data

**Table:**

C_16_H_10_FNO_3_	*D*_x_ = 1.543 Mg m^−^^3^
*M**_r_* = 283.25	Melting point: 432 K
Monoclinic, *P*2_1_/*n*	Mo *K*α radiation, λ = 0.71073 Å
*a* = 3.7966 (2) Å	Cell parameters from 1976 reflections
*b* = 24.8289 (11) Å	θ = 2.0–26.0°
*c* = 12.9470 (7) Å	µ = 0.12 mm^−^^1^
β = 92.870 (2)°	*T* = 296 K
*V* = 1218.92 (11) Å^3^	Prism, colorless
*Z* = 4	0.32 × 0.25 × 0.21 mm
*F*(000) = 584	

### N-(2-Fluorophenyl)-2-oxo-2H-chromene-3-carboxamide (I) Data collection

**Table:**

Bruker SMART APEXII CCD diffractometer	2154 independent reflections
Radiation source: fine-focus sealed tube	1976 reflections with *I* > 2σ(*I*)
Detector resolution: 1.09 pixels mm^-1^	*R*_int_ = 0.076
φ and Ω scans	θ_max_ = 25.0°, θ_min_ = 3.2°
Absorption correction: multi-scan (SADABS; Krause *et al.*, 2015)	*h* = −4→4
*T*_min_ = 0.963, *T*_max_ = 0.974	*k* = −29→29
22396 measured reflections	*l* = −15→15

### N-(2-Fluorophenyl)-2-oxo-2H-chromene-3-carboxamide (I) Refinement

**Table:**

Refinement on *F*^2^	Primary atom site location: structure-invariant direct methods
Least-squares matrix: full	Secondary atom site location: difference Fourier map
*R*[*F*^2^ > 2σ(*F*^2^)] = 0.093	Hydrogen site location: inferred from neighbouring sites
*wR*(*F*^2^) = 0.149	H-atom parameters constrained
*S* = 1.28	*w* = 1/[σ^2^(*F*_o_^2^) + 3.8827*P*] where *P* = (*F*_o_^2^ + 2*F*_c_^2^)/3
2154 reflections	(Δ/σ)_max_ < 0.001
190 parameters	Δρ_max_ = 0.27 e Å^−^^3^
0 restraints	Δρ_min_ = −0.35 e Å^−^^3^

### N-(2-Fluorophenyl)-2-oxo-2H-chromene-3-carboxamide (I) Special details

**Table:**

Geometry. All esds (except the esd in the dihedral angle between two l.s. planes) are estimated using the full covariance matrix. The cell esds are taken into account individually in the estimation of esds in distances, angles and torsion angles; correlations between esds in cell parameters are only used when they are defined by crystal symmetry. An approximate (isotropic) treatment of cell esds is used for estimating esds involving l.s. planes.

### N-(2-Fluorophenyl)-2-oxo-2H-chromene-3-carboxamide (I) Fractional atomic coordinates and isotropic or equivalent isotropic displacement parameters (Å^2^)

**Table:**

	*x*	*y*	*z*	*U*_iso_*/*U*_eq_	
O1	0.1241 (8)	0.90012 (11)	0.6794 (2)	0.0246 (7)	
O2	−0.1271 (7)	0.97653 (10)	0.63588 (19)	0.0186 (6)	
F1	0.4305 (7)	0.78279 (10)	0.79180 (18)	0.0333 (7)	
O3	−0.1113 (8)	0.92419 (11)	0.9918 (2)	0.0231 (7)	
C4	−0.3668 (11)	1.05905 (16)	0.5771 (3)	0.0194 (9)	
H4	−0.308278	1.050165	0.510313	0.023*	
N1	0.1219 (9)	0.86595 (12)	0.8778 (2)	0.0182 (7)	
H1	0.159091	0.862348	0.813205	0.022*	
C3	−0.2944 (10)	1.02407 (16)	0.6582 (3)	0.0190 (9)	
C8	−0.3803 (10)	1.03577 (15)	0.7596 (3)	0.0157 (8)	
C2	−0.0334 (11)	0.93910 (16)	0.7095 (3)	0.0190 (9)	
C6	−0.6140 (11)	1.12069 (16)	0.6980 (3)	0.0226 (9)	
H6	−0.718705	1.153697	0.710989	0.027*	
C9	−0.2900 (10)	0.99706 (16)	0.8372 (3)	0.0168 (8)	
H9	−0.346070	1.004073	0.905013	0.020*	
C12	0.3930 (11)	0.77959 (17)	0.8957 (3)	0.0218 (9)	
C10	−0.0380 (11)	0.91219 (16)	0.9044 (3)	0.0190 (9)	
C11	0.2356 (10)	0.82297 (16)	0.9418 (3)	0.0176 (9)	
C7	−0.5444 (10)	1.08505 (16)	0.7777 (3)	0.0203 (9)	
H7	−0.606932	1.093797	0.844139	0.024*	
C15	0.3174 (11)	0.77538 (16)	1.1028 (3)	0.0227 (9)	
H15	0.291815	0.773906	1.173785	0.027*	
C1	−0.1257 (11)	0.95043 (15)	0.8160 (3)	0.0169 (8)	
C16	0.1984 (10)	0.82015 (16)	1.0487 (3)	0.0184 (9)	
H16	0.093706	0.848377	1.083041	0.022*	
C5	−0.5277 (11)	1.10740 (16)	0.5972 (3)	0.0215 (9)	
H5	−0.579398	1.131395	0.543369	0.026*	
C13	0.5118 (11)	0.73507 (17)	0.9487 (3)	0.0266 (10)	
H13	0.616607	0.706863	0.914296	0.032*	
C14	0.4739 (11)	0.73258 (17)	1.0543 (3)	0.0254 (10)	
H14	0.552215	0.702649	1.092051	0.031*	

### N-(2-Fluorophenyl)-2-oxo-2H-chromene-3-carboxamide (I) Atomic displacement parameters (Å^2^)

**Table:**

	*U*^11^	*U*^22^	*U*^33^	*U*^12^	*U*^13^	*U*^23^
O1	0.0380 (18)	0.0246 (16)	0.0119 (14)	0.0102 (14)	0.0068 (12)	−0.0017 (12)
O2	0.0253 (16)	0.0207 (14)	0.0100 (13)	0.0006 (12)	0.0032 (11)	−0.0003 (11)
F1	0.0473 (17)	0.0349 (15)	0.0178 (13)	0.0155 (13)	0.0019 (11)	−0.0038 (11)
O3	0.0350 (18)	0.0233 (15)	0.0117 (14)	0.0047 (13)	0.0070 (12)	−0.0004 (12)
C4	0.023 (2)	0.024 (2)	0.0112 (19)	−0.0067 (18)	−0.0020 (16)	−0.0005 (16)
N1	0.0257 (19)	0.0216 (18)	0.0075 (16)	0.0017 (15)	0.0013 (14)	0.0013 (13)
C3	0.016 (2)	0.021 (2)	0.020 (2)	−0.0024 (16)	0.0017 (17)	−0.0027 (17)
C8	0.016 (2)	0.019 (2)	0.0120 (19)	−0.0060 (16)	−0.0010 (15)	−0.0041 (15)
C2	0.022 (2)	0.020 (2)	0.014 (2)	−0.0042 (18)	−0.0001 (17)	0.0000 (17)
C6	0.024 (2)	0.018 (2)	0.025 (2)	0.0008 (18)	0.0008 (18)	−0.0001 (17)
C9	0.015 (2)	0.024 (2)	0.0118 (18)	−0.0064 (17)	0.0041 (15)	−0.0009 (16)
C12	0.023 (2)	0.027 (2)	0.015 (2)	−0.0007 (18)	−0.0014 (17)	−0.0060 (17)
C10	0.022 (2)	0.023 (2)	0.013 (2)	−0.0030 (17)	0.0037 (16)	−0.0015 (16)
C11	0.015 (2)	0.021 (2)	0.016 (2)	−0.0035 (16)	−0.0016 (16)	−0.0006 (16)
C7	0.019 (2)	0.025 (2)	0.017 (2)	−0.0042 (17)	0.0021 (16)	−0.0046 (17)
C15	0.022 (2)	0.028 (2)	0.018 (2)	−0.0017 (18)	0.0039 (17)	0.0043 (17)
C1	0.018 (2)	0.019 (2)	0.0135 (19)	−0.0034 (16)	0.0004 (16)	−0.0016 (15)
C16	0.017 (2)	0.021 (2)	0.017 (2)	−0.0014 (17)	0.0018 (16)	−0.0019 (16)
C5	0.022 (2)	0.023 (2)	0.020 (2)	−0.0014 (17)	0.0006 (17)	0.0043 (17)
C13	0.029 (2)	0.018 (2)	0.032 (3)	0.0028 (18)	−0.004 (2)	−0.0064 (19)
C14	0.025 (2)	0.022 (2)	0.029 (2)	−0.0048 (18)	−0.0096 (19)	0.0069 (18)

### N-(2-Fluorophenyl)-2-oxo-2H-chromene-3-carboxamide (I) Geometric parameters (Å, º)

**Table:**

O1—C2	1.212 (5)	C6—H6	0.9300
O2—C2	1.365 (5)	C9—C1	1.350 (5)
O2—C3	1.378 (5)	C9—H9	0.9300
F1—C12	1.363 (5)	C12—C13	1.366 (6)
O3—C10	1.215 (5)	C12—C11	1.382 (5)
C4—C5	1.378 (6)	C10—C1	1.512 (5)
C4—C3	1.380 (5)	C11—C16	1.399 (5)
C4—H4	0.9300	C7—H7	0.9300
N1—C10	1.352 (5)	C15—C16	1.378 (6)
N1—C11	1.406 (5)	C15—C14	1.383 (6)
N1—H1	0.8600	C15—H15	0.9300
C3—C8	1.398 (5)	C16—H16	0.9300
C8—C7	1.398 (6)	C5—H5	0.9300
C8—C9	1.420 (5)	C13—C14	1.383 (6)
C2—C1	1.467 (5)	C13—H13	0.9300
C6—C7	1.375 (6)	C14—H14	0.9300
C6—C5	1.401 (6)		
			
C2—O2—C3	122.9 (3)	O3—C10—N1	124.9 (4)
C5—C4—C3	118.6 (4)	O3—C10—N1	124.9 (4)
C5—C4—H4	120.7	O3—C10—C1	119.9 (4)
C3—C4—H4	120.7	O3—C10—C1	119.9 (4)
C10—N1—C11	128.5 (3)	O3—C10—C1	119.9 (4)
C10—N1—H1	115.7	N1—C10—C1	115.2 (3)
C11—N1—H1	115.7	C12—C11—C16	117.1 (4)
O2—C3—C4	117.1 (3)	C12—C11—N1	117.6 (3)
O2—C3—C8	120.4 (3)	C16—C11—N1	125.3 (4)
C4—C3—C8	122.5 (4)	C6—C7—C8	120.5 (4)
C7—C8—C3	117.8 (4)	C6—C7—H7	119.7
C7—C8—C9	124.6 (3)	C8—C7—H7	119.7
C3—C8—C9	117.7 (4)	C16—C15—C14	121.8 (4)
O1—C2—O1	0.0 (3)	C16—C15—H15	119.1
O1—C2—O2	115.8 (3)	C14—C15—H15	119.1
O1—C2—O2	115.8 (3)	C9—C1—C2	119.4 (4)
O1—C2—C1	126.9 (4)	C9—C1—C10	118.1 (3)
O1—C2—C1	126.9 (4)	C2—C1—C10	122.5 (3)
O2—C2—C1	117.3 (3)	C15—C16—C11	119.8 (4)
C7—C6—C5	120.2 (4)	C15—C16—H16	120.1
C7—C6—H6	119.9	C11—C16—H16	120.1
C5—C6—H6	119.9	C4—C5—C6	120.5 (4)
C1—C9—C8	122.3 (3)	C4—C5—H5	119.7
C1—C9—H9	118.9	C6—C5—H5	119.7
C8—C9—H9	118.9	C12—C13—C14	119.0 (4)
F1—C12—C13	119.7 (4)	C12—C13—H13	120.5
F1—C12—C13	119.7 (4)	C14—C13—H13	120.5
F1—C12—C11	116.7 (4)	C15—C14—C13	118.8 (4)
F1—C12—C11	116.7 (4)	C15—C14—H14	120.6
C13—C12—C11	123.6 (4)	C13—C14—H14	120.6
O3—C10—N1	124.9 (4)		
			
C2—O2—C3—C4	178.5 (4)	C8—C9—C1—C2	0.5 (6)
C2—O2—C3—C8	−0.8 (5)	C8—C9—C1—C10	179.4 (4)
C5—C4—C3—O2	−178.8 (4)	O1—C2—C1—C9	177.3 (4)
C5—C4—C3—C8	0.4 (6)	O1—C2—C1—C9	177.3 (4)
O2—C3—C8—C7	178.9 (3)	O2—C2—C1—C9	−1.3 (6)
C4—C3—C8—C7	−0.2 (6)	O1—C2—C1—C10	−1.6 (7)
O2—C3—C8—C9	−0.2 (6)	O1—C2—C1—C10	−1.6 (7)
C4—C3—C8—C9	−179.4 (4)	O2—C2—C1—C10	179.8 (3)
C3—O2—C2—O1	−177.3 (4)	O3—C10—C1—C9	−1.0 (6)
C3—O2—C2—O1	−177.3 (4)	O3—C10—C1—C9	−1.0 (6)
C3—O2—C2—C1	1.5 (5)	O3—C10—C1—C9	−1.0 (6)
C7—C8—C9—C1	−178.8 (4)	N1—C10—C1—C9	179.1 (4)
C3—C8—C9—C1	0.3 (6)	O3—C10—C1—C2	177.9 (4)
F1—F1—C12—C13	0.0 (16)	O3—C10—C1—C2	177.9 (4)
C11—N1—C10—C1	179.9 (4)	O3—C10—C1—C2	177.9 (4)
F1—C12—C11—C16	−179.6 (4)	N1—C10—C1—C2	−2.0 (6)
F1—C12—C11—C16	−179.6 (4)	C14—C15—C16—C11	−0.1 (6)
C13—C12—C11—C16	−0.1 (6)	C12—C11—C16—C15	0.1 (6)
F1—C12—C11—N1	1.2 (5)	N1—C11—C16—C15	179.2 (4)
F1—C12—C11—N1	1.2 (5)	C3—C4—C5—C6	0.3 (6)
C13—C12—C11—N1	−179.3 (4)	C7—C6—C5—C4	−1.2 (6)
C10—N1—C11—C12	179.8 (4)	F1—C12—C13—C14	179.6 (4)
C10—N1—C11—C16	0.7 (7)	F1—C12—C13—C14	179.6 (4)
C5—C6—C7—C8	1.4 (6)	C11—C12—C13—C14	0.1 (7)
C3—C8—C7—C6	−0.7 (6)	C16—C15—C14—C13	0.1 (6)
C9—C8—C7—C6	178.4 (4)	C12—C13—C14—C15	−0.1 (6)

### N-(2-Fluorophenyl)-2-oxo-2H-chromene-3-carboxamide (I) Hydrogen-bond geometry (Å, º)

**Table:**

*D*—H···*A*	*D*—H	H···*A*	*D*···*A*	*D*—H···*A*
N1—H1···O1	0.86	1.97	2.706 (4)	143
N1—H1···F1	0.86	2.25	2.647 (4)	108
C9—H9···O3	0.93	2.43	2.757 (5)	101
C16—H16···O3	0.93	2.34	2.918 (5)	120
C7—H7···O3^i^	0.93	2.47	3.326 (5)	154
C15—H15···F1^ii^	0.93	2.53	3.255 (5)	135

Symmetry codes: (i) −*x*−1, −*y*+2, −*z*+2; (ii) *x*−1/2, −*y*+3/2, *z*+1/2.

### N-[4-(Methylsulfonyl)phenyl]-2-oxo-2H-chromene-3-carboxamide (II) Crystal data

**Table:**

C_17_H_13_NO_5_S	*F*(000) = 712
*M**_r_* = 343.36	*D*_x_ = 1.500 Mg m^−^^3^
Monoclinic, *P*2_1_/*c*	Melting point: 458 K
Hall symbol: -P 2ybc	Mo *K*α radiation, λ = 0.71073 Å
*a* = 17.2099 (18) Å	Cell parameters from 2177 reflections
*b* = 7.0495 (7) Å	θ = 3.0–26.0°
*c* = 12.6535 (12) Å	µ = 0.24 mm^−^^1^
β = 98.022 (3)°	*T* = 289 K
*V* = 1520.1 (3) Å^3^	Prism, colourless
*Z* = 4	0.38 × 0.30 × 0.25 mm

### N-[4-(Methylsulfonyl)phenyl]-2-oxo-2H-chromene-3-carboxamide (II) Data collection

**Table:**

Bruker SMART APEXII CCD diffractometer	3042 independent reflections
Radiation source: fine-focus sealed tube	2177 reflections with *I* > 2σ(*I*)
Graphite monochromator	*R*_int_ = 0.090
Detector resolution: 1.09 pixels mm^-1^	θ_max_ = 26.2°, θ_min_ = 3.1°
φ and Ω scans	*h* = −21→21
Absorption correction: multi-scan (SADABS; Krause *et al.*, 2015)	*k* = −8→8
*T*_min_ = 0.915, *T*_max_ = 0.941	*l* = −15→15
19137 measured reflections	

### N-[4-(Methylsulfonyl)phenyl]-2-oxo-2H-chromene-3-carboxamide (II) Refinement

**Table:**

Refinement on *F*^2^	Primary atom site location: structure-invariant direct methods
Least-squares matrix: full	Secondary atom site location: difference Fourier map
*R*[*F*^2^ > 2σ(*F*^2^)] = 0.064	Hydrogen site location: mixed
*wR*(*F*^2^) = 0.156	H atoms treated by a mixture of independent and constrained refinement
*S* = 1.05	*w* = 1/[σ^2^(*F*_o_^2^) + (0.0533*P*)^2^ + 1.6684*P*] where *P* = (*F*_o_^2^ + 2*F*_c_^2^)/3
3042 reflections	(Δ/σ)_max_ < 0.001
221 parameters	Δρ_max_ = 0.26 e Å^−^^3^
0 restraints	Δρ_min_ = −0.42 e Å^−^^3^
0 constraints	

### N-[4-(Methylsulfonyl)phenyl]-2-oxo-2H-chromene-3-carboxamide (II) Special details

**Table:**

Geometry. All esds (except the esd in the dihedral angle between two l.s. planes) are estimated using the full covariance matrix. The cell esds are taken into account individually in the estimation of esds in distances, angles and torsion angles; correlations between esds in cell parameters are only used when they are defined by crystal symmetry. An approximate (isotropic) treatment of cell esds is used for estimating esds involving l.s. planes.

### N-[4-(Methylsulfonyl)phenyl]-2-oxo-2H-chromene-3-carboxamide (II) Fractional atomic coordinates and isotropic or equivalent isotropic displacement parameters (Å^2^)

**Table:**

	*x*	*y*	*z*	*U*_iso_*/*U*_eq_	
S1	0.40542 (5)	0.18990 (15)	0.37569 (8)	0.0515 (3)	
O2	−0.17185 (13)	0.0829 (3)	0.13403 (17)	0.0435 (6)	
O1	−0.06266 (14)	0.0440 (4)	0.24183 (18)	0.0537 (7)	
O3	0.06755 (14)	0.2706 (4)	0.01234 (19)	0.0552 (7)	
O4	0.40545 (16)	0.1716 (5)	0.4883 (2)	0.0679 (8)	
N1	0.07441 (17)	0.1345 (4)	0.1762 (2)	0.0393 (7)	
C2	−0.09242 (19)	0.1004 (5)	0.1545 (3)	0.0406 (8)	
C9	−0.09491 (19)	0.2534 (5)	−0.0178 (2)	0.0377 (7)	
H9	−0.069180	0.310057	−0.069533	0.045*	
C10	0.03591 (19)	0.2021 (4)	0.0840 (2)	0.0370 (7)	
C14	0.30808 (19)	0.1684 (5)	0.3125 (3)	0.0397 (8)	
C12	0.17416 (19)	0.1029 (5)	0.3246 (2)	0.0399 (8)	
H12	0.135220	0.066486	0.364521	0.048*	
C8	−0.17861 (19)	0.2407 (4)	−0.0351 (2)	0.0373 (7)	
C3	−0.21538 (19)	0.1513 (5)	0.0418 (3)	0.0385 (7)	
C15	0.2889 (2)	0.2111 (5)	0.2049 (3)	0.0441 (8)	
H15	0.328076	0.247507	0.165326	0.053*	
C5	−0.3410 (2)	0.1977 (6)	−0.0567 (3)	0.0547 (10)	
H5	−0.395167	0.182412	−0.065105	0.066*	
C1	−0.05228 (18)	0.1853 (4)	0.0718 (2)	0.0342 (7)	
C4	−0.2957 (2)	0.1282 (5)	0.0328 (3)	0.0476 (9)	
H4	−0.318471	0.067130	0.085906	0.057*	
O5	0.43786 (17)	0.3599 (5)	0.3370 (3)	0.0790 (10)	
C16	0.21218 (19)	0.1998 (5)	0.1565 (3)	0.0414 (8)	
H16	0.199419	0.226799	0.084194	0.050*	
C6	−0.3062 (2)	0.2909 (6)	−0.1347 (3)	0.0544 (10)	
H6	−0.337561	0.340156	−0.194251	0.065*	
C11	0.15400 (18)	0.1474 (4)	0.2169 (2)	0.0347 (7)	
C13	0.25051 (19)	0.1120 (5)	0.3723 (3)	0.0408 (8)	
H13	0.263593	0.080650	0.443957	0.049*	
C7	−0.2264 (2)	0.3113 (5)	−0.1252 (3)	0.0473 (9)	
H7	−0.203869	0.372142	−0.178679	0.057*	
C17	0.4539 (3)	−0.0076 (8)	0.3316 (4)	0.0875 (16)	
H17A	0.454577	0.001883	0.256090	0.131*	
H17B	0.506822	−0.011464	0.367707	0.131*	
H17C	0.426947	−0.121333	0.347035	0.131*	
H1	0.043 (2)	0.093 (5)	0.217 (3)	0.046 (10)*	

### N-[4-(Methylsulfonyl)phenyl]-2-oxo-2H-chromene-3-carboxamide (II) Atomic displacement parameters (Å^2^)

**Table:**

	*U*^11^	*U*^22^	*U*^33^	*U*^12^	*U*^13^	*U*^23^
S1	0.0364 (5)	0.0667 (6)	0.0521 (6)	−0.0024 (4)	0.0093 (4)	−0.0055 (5)
O2	0.0400 (13)	0.0528 (14)	0.0387 (12)	−0.0058 (11)	0.0090 (10)	0.0089 (11)
O1	0.0497 (15)	0.0725 (18)	0.0387 (13)	−0.0077 (13)	0.0054 (11)	0.0189 (13)
O3	0.0475 (14)	0.0728 (18)	0.0483 (14)	0.0010 (13)	0.0171 (12)	0.0199 (13)
O4	0.0531 (16)	0.102 (2)	0.0483 (15)	0.0026 (16)	0.0050 (12)	−0.0052 (16)
N1	0.0397 (15)	0.0412 (16)	0.0381 (15)	−0.0028 (13)	0.0097 (12)	0.0061 (13)
C2	0.0439 (19)	0.0399 (18)	0.0390 (18)	−0.0052 (15)	0.0101 (15)	−0.0012 (16)
C9	0.0453 (18)	0.0365 (17)	0.0336 (16)	−0.0019 (14)	0.0131 (14)	−0.0010 (14)
C10	0.0474 (19)	0.0305 (16)	0.0354 (17)	0.0017 (15)	0.0138 (14)	−0.0012 (14)
C14	0.0363 (17)	0.0390 (18)	0.0454 (18)	0.0021 (14)	0.0112 (14)	−0.0008 (15)
C12	0.0411 (18)	0.0428 (18)	0.0383 (17)	−0.0020 (15)	0.0152 (14)	0.0025 (15)
C8	0.0450 (18)	0.0300 (16)	0.0376 (17)	0.0015 (14)	0.0082 (14)	−0.0030 (14)
C3	0.0405 (18)	0.0366 (17)	0.0375 (17)	−0.0010 (14)	0.0027 (14)	−0.0006 (14)
C15	0.0437 (19)	0.047 (2)	0.0453 (19)	0.0012 (16)	0.0200 (15)	0.0069 (16)
C5	0.041 (2)	0.057 (2)	0.065 (2)	−0.0015 (18)	0.0013 (18)	−0.002 (2)
C1	0.0400 (17)	0.0309 (16)	0.0334 (16)	−0.0007 (14)	0.0111 (13)	−0.0015 (13)
C4	0.045 (2)	0.047 (2)	0.052 (2)	−0.0032 (16)	0.0116 (16)	0.0039 (17)
O5	0.0609 (18)	0.090 (2)	0.087 (2)	−0.0316 (17)	0.0160 (16)	0.0050 (18)
C16	0.0447 (19)	0.0444 (19)	0.0374 (17)	0.0036 (16)	0.0140 (14)	0.0048 (16)
C6	0.053 (2)	0.057 (2)	0.050 (2)	0.0068 (19)	−0.0047 (17)	0.0033 (19)
C11	0.0389 (17)	0.0288 (16)	0.0371 (17)	0.0024 (13)	0.0083 (13)	−0.0014 (13)
C13	0.0422 (18)	0.0462 (19)	0.0353 (17)	0.0046 (15)	0.0102 (14)	0.0020 (15)
C7	0.055 (2)	0.048 (2)	0.0394 (18)	0.0050 (18)	0.0085 (16)	0.0050 (16)
C17	0.050 (3)	0.121 (4)	0.089 (3)	0.027 (3)	0.001 (2)	−0.031 (3)

### N-[4-(Methylsulfonyl)phenyl]-2-oxo-2H-chromene-3-carboxamide (II) Geometric parameters (Å, º)

**Table:**

S1—O4	1.431 (3)	C12—C11	1.394 (4)
S1—O4	1.431 (3)	C12—H12	0.9300
S1—O5	1.437 (3)	C8—C3	1.385 (4)
S1—C17	1.754 (5)	C8—C7	1.401 (5)
S1—C14	1.759 (3)	C3—C4	1.380 (5)
O2—C2	1.361 (4)	C15—C16	1.379 (5)
O2—C3	1.382 (4)	C15—H15	0.9300
O1—C2	1.218 (4)	C5—C4	1.372 (5)
O3—C10	1.221 (4)	C5—C6	1.389 (5)
N1—C10	1.345 (4)	C5—H5	0.9300
N1—C11	1.397 (4)	C4—H4	0.9300
N1—H1	0.86 (4)	C16—C11	1.392 (4)
C2—C1	1.460 (4)	C16—H16	0.9300
C9—C1	1.350 (4)	C6—C7	1.370 (5)
C9—C8	1.429 (4)	C6—H6	0.9300
C9—H9	0.9300	C13—H13	0.9300
C10—C1	1.509 (4)	C7—H7	0.9300
C14—C13	1.386 (4)	C17—H17A	0.9600
C14—C15	1.389 (5)	C17—H17B	0.9600
C12—C13	1.369 (4)	C17—H17C	0.9600
			
O4—S1—O5	117.9 (2)	C4—C3—O2	116.6 (3)
O4—S1—O5	117.9 (2)	C4—C3—C8	123.1 (3)
O4—S1—C17	108.1 (2)	O2—C3—C8	120.3 (3)
O4—S1—C17	108.1 (2)	C16—C15—C14	120.3 (3)
O5—S1—C17	109.2 (2)	C16—C15—H15	119.8
O4—S1—C14	108.22 (16)	C14—C15—H15	119.8
O4—S1—C14	108.22 (16)	C4—C5—C6	120.3 (3)
O5—S1—C14	107.94 (17)	C4—C5—H5	119.9
C17—S1—C14	104.63 (19)	C6—C5—H5	119.9
C2—O2—C3	122.7 (3)	C9—C1—C2	119.5 (3)
C10—N1—C11	129.5 (3)	C9—C1—C10	118.6 (3)
C10—N1—H1	112 (2)	C2—C1—C10	121.9 (3)
C11—N1—H1	118 (2)	C5—C4—C3	118.2 (3)
O1—C2—O2	115.3 (3)	C5—C4—H4	120.9
O1—C2—O2	115.3 (3)	C3—C4—H4	120.9
O1—C2—C1	127.1 (3)	C15—C16—C11	119.3 (3)
O1—C2—C1	127.1 (3)	C15—C16—H16	120.4
O2—C2—C1	117.6 (3)	C11—C16—H16	120.4
C1—C9—C8	121.6 (3)	C7—C6—C5	120.9 (3)
C1—C9—H9	119.2	C7—C6—H6	119.6
C8—C9—H9	119.2	C5—C6—H6	119.6
O3—C10—N1	124.6 (3)	C16—C11—C12	119.8 (3)
O3—C10—N1	124.6 (3)	C16—C11—N1	123.9 (3)
O3—C10—N1	124.6 (3)	C12—C11—N1	116.3 (3)
O3—C10—C1	120.1 (3)	C12—C13—C14	119.2 (3)
O3—C10—C1	120.1 (3)	C12—C13—H13	120.4
O3—C10—C1	120.1 (3)	C14—C13—H13	120.4
N1—C10—C1	115.3 (3)	C6—C7—C8	120.1 (3)
C13—C14—C15	120.5 (3)	C6—C7—H7	119.9
C13—C14—S1	119.0 (3)	C8—C7—H7	119.9
C15—C14—S1	120.5 (2)	S1—C17—H17A	109.5
C13—C12—C11	120.9 (3)	S1—C17—H17B	109.5
C13—C12—H12	119.6	H17A—C17—H17B	109.5
C11—C12—H12	119.6	S1—C17—H17C	109.5
C3—C8—C7	117.4 (3)	H17A—C17—H17C	109.5
C3—C8—C9	118.1 (3)	H17B—C17—H17C	109.5
C7—C8—C9	124.5 (3)		
			
C3—O2—C2—O1	175.6 (3)	O2—C2—C1—C9	4.1 (5)
C3—O2—C2—O1	175.6 (3)	O1—C2—C1—C10	4.0 (5)
C3—O2—C2—C1	−3.6 (5)	O1—C2—C1—C10	4.0 (5)
C11—N1—C10—O3	9.0 (6)	O2—C2—C1—C10	−176.9 (3)
C11—N1—C10—O3	9.0 (6)	O3—C10—C1—C9	−2.4 (5)
C11—N1—C10—O3	9.0 (6)	O3—C10—C1—C9	−2.4 (5)
C11—N1—C10—C1	−172.1 (3)	O3—C10—C1—C9	−2.4 (5)
O4—S1—C14—C13	−9.2 (3)	N1—C10—C1—C9	178.7 (3)
O4—S1—C14—C13	−9.2 (3)	O3—C10—C1—C2	178.6 (3)
O5—S1—C14—C13	−137.9 (3)	O3—C10—C1—C2	178.6 (3)
C17—S1—C14—C13	105.9 (3)	O3—C10—C1—C2	178.6 (3)
O4—S1—C14—C15	169.4 (3)	N1—C10—C1—C2	−0.3 (4)
O4—S1—C14—C15	169.4 (3)	C6—C5—C4—C3	−0.9 (6)
O5—S1—C14—C15	40.8 (3)	O2—C3—C4—C5	178.7 (3)
C17—S1—C14—C15	−75.5 (3)	C8—C3—C4—C5	−0.1 (5)
C1—C9—C8—C3	−1.7 (5)	C14—C15—C16—C11	0.8 (5)
C1—C9—C8—C7	178.8 (3)	C4—C5—C6—C7	1.5 (6)
C2—O2—C3—C4	−178.4 (3)	C15—C16—C11—C12	−1.5 (5)
C2—O2—C3—C8	0.5 (5)	C15—C16—C11—N1	179.0 (3)
C7—C8—C3—C4	0.5 (5)	C13—C12—C11—C16	0.8 (5)
C9—C8—C3—C4	−179.0 (3)	C13—C12—C11—N1	−179.7 (3)
C7—C8—C3—O2	−178.2 (3)	C10—N1—C11—C16	−14.9 (5)
C9—C8—C3—O2	2.3 (5)	C10—N1—C11—C12	165.6 (3)
C13—C14—C15—C16	0.7 (5)	C11—C12—C13—C14	0.7 (5)
S1—C14—C15—C16	−178.0 (3)	C15—C14—C13—C12	−1.4 (5)
C8—C9—C1—C2	−1.5 (5)	S1—C14—C13—C12	177.2 (3)
C8—C9—C1—C10	179.4 (3)	C5—C6—C7—C8	−1.1 (6)
O1—C2—C1—C9	−175.0 (3)	C3—C8—C7—C6	0.1 (5)
O1—C2—C1—C9	−175.0 (3)	C9—C8—C7—C6	179.6 (3)

### N-[4-(Methylsulfonyl)phenyl]-2-oxo-2H-chromene-3-carboxamide (II) Hydrogen-bond geometry (Å, º)

*Cg*1 and *Cg*2 are the centroids of the O2/C2/C1/C9/C8/C3 and C3/C4/C5/C6/C7/C8 rings.

**Table:**

*D*—H···*A*	*D*—H	H···*A*	*D*···*A*	*D*—H···*A*
N1—H1···O1	0.86 (4)	1.91 (4)	2.685 (4)	149 (3)
C9—H9···O3	0.93	2.45	2.771 (4)	100
C16—H16···O3	0.93	2.34	2.916 (4)	119
C13—H13···O4	0.93	2.51	2.890 (4)	105
C7—H7···O1^i^	0.93	2.82	3.624 (4)	146
C17—H17*A*···O5^ii^	0.96	2.53	3.163 (5)	123
C17—H17*B*···O4^iii^	0.96	2.47	3.293 (5)	144
C12—H12···*Cg*1^iv^	0.93	2.97	3.509 (4)	118
C13—H13···*Cg*2^iv^	0.93	2.86	3.522 (4)	129

Symmetry codes: (i) *x*, −*y*+1/2, *z*−1/2; (ii) −*x*+1, *y*−1/2, −*z*+1/2; (iii) −*x*+1, −*y*, −*z*+1; (iv) −*x*, *y*−1/2, −*z*+1/2.
